# Predictors and Mortality of Rapidly Progressive Interstitial Lung Disease in Patients With Idiopathic Inflammatory Myopathy: A Series of 474 Patients

**DOI:** 10.3389/fmed.2020.00363

**Published:** 2020-07-31

**Authors:** Yuhui Li, Xiaojuan Gao, Yimin Li, Xiaohui Jia, Xuewu Zhang, Yan Xu, Yuzhou Gan, Shiming Li, Renli Chen, Jing He, Xiaolin Sun

**Affiliations:** ^1^Beijing Key Laboratory for Rheumatism and Immune Diagnosis (BZ0135), Department of Rheumatology and Immunology, Peking University People's Hospital, Beijing, China; ^2^Department of Rheumatology, Ningde Hospital, Affiliated Hospital of Fujian Medical University, Ningde, China; ^3^Department of Rheumatology, The First Hospital of Hebei Medical University, Shijiazhuang, China; ^4^Department of Neurology, Peking University People's Hospital, Beijing, China; ^5^Department of Endocrinology, People's Hospital of Wushan County, Gansu, China

**Keywords:** myositis, interstitial lung disease, MSAs, rapidly progressive, survival

## Abstract

**Objective:** This study was conducted to identify the characteristics and prognosis of rapidly progressive interstitial lung disease (RP-ILD) in idiopathic inflammatory myopathy (IIM) and to assess the predictors for poor survival of RP-ILD in IIM.

**Methods:** A total of 474 patients with IIM were enrolled retrospectively according to medical records from Peking University People's Hospital. Clinical and laboratory characteristics recorded at the diagnosis of patients with RP-ILD and chronic ILD (C-ILD) were compared. The Kaplan–Meier estimator and univariate and multivariate analyses were used for data analysis.

**Results:** ILD was identified in 65% (308/474) of patients with IIM. Patients with ILD were classified into two groups based on lung features: RP-ILD (38%, 117/308) and C-ILD (62%, 191/308). RP-ILD resulted in significantly higher mortality in IIM compared with C-ILD (27.4 vs. 7.9%, *P* < 0.05). In this study, by comparing IIM patients with and without RP-ILD, a list of initial predictors for RP-ILD development were identified, which included older age at onset, decreased peripheral lymphocytes, skin involvement (periungual erythema, skin ulceration, and subcutaneous/mediastinal emphysema), presence of anti-MDA5 antibody, serum tumor markers, etc. Further multivariate Cox proportional hazards model analysis identified that anti-MDA5 positivity was an independent risk factor for mortality due to RP-ILD (*P* < 0.05), and lymphocytes <30% in BALF might also be associated with poor survival of myositis-associated RP-ILD (*P* < 0.05).

**Conclusion:** Our study shows that RP-ILD results in increased mortality in IIM. Anti-MDA5 positivity and a lower lymphocyte ratio in BALF might be the predictive factor of mortality due to RP-ILD.

## Introduction

Idiopathic inflammatory myopathy (IIM) is a group of systemic autoimmune diseases characterized by skin rash, proximal muscle weakness, and extramuscular manifestations, such as arthralgia, fever, and interstitial lung disease (ILD). Dermatomyositis (DM), polymyositis (PM), and clinically amyopathic dermatomyositis (CADM) are the three main subtypes of IIM (1, 2). Myositis-associated ILD is one of the leading extramuscular features, occurring in 20–80% of all PM/DM/CADM patients (3, 4). Rapidly progressive ILD (RP-ILD) in IIM is a life-threatening subtype of myositis-associated ILD, which tends to be resistant to high-dose glucocorticoid treatment and immunosuppressants (4–6). Recently, a study in a European myositis cohort reported that 40–60% of patients with RP-ILD were admitted to the ICU, and hospital mortality was 45–51% (7). Some patients with RP-ILD decline within weeks, but for other patients, the time to ILD-induced deterioration is on the order of years (8), and the 5-year survival rate is more than 85% in myositis-associated ILD (9, 10). However, it is difficult to predict whether patients with myositis-associated ILD will develop fatal disease progression at the early stage of the disease. Therefore, it is necessary to identify potential factors to predict survival of patients with myositis-associated RP-ILD in the early stage of disease development.

The pathogenesis of lung injury in myositis is unclear. Although anti-aminoacyl tRNA synthetase (ARS) and anti-melanoma differentiation-associated 5 (MDA5) antibodies have been described as associated with RP-ILD (11), the exact pathophysiology and diagnostic value of these autoantibodies remain to be elucidated. Previous studies have reported the relationship between poor outcomes of RP-ILD with DM classification, older age, skin ulceration, lack of myositis, and positivity of anti-MDA5 antibody (12–14). Fever, elevated serum CRP, and ferritin levels and ground-glass attenuation on high-resolution CT (HRCT) have been suggested as risk factors for ILD in myositis (14–16). However, due to the heterogeneity of IIM, the prevalence, risk predictors, and survival rates of RP-ILD vary widely among different studies.

In this study, we investigated the clinical and laboratory characteristics at the time of diagnosis of ILD in DM/PM/CADM patients. Moreover, we compared serum biomarkers and pulmonary characteristics of RP-ILD and chronic-ILD (C-ILD) to exploit potential prognostic markers of myositis-associated RP-ILD in a large-scale patient cohort in China.

## Materials and Methods

### Patients

Patients diagnosed with DM/PM/CADM in the department of rheumatology and immunology, Peking University People's Hospital between July 2000 and October 2019 were identified in this retrospective study. Cases satisfied diagnostic criteria suggested by the Bohan & Peter DM/PM classification or Sontheimer's definitions (2, 17). CADM is the combination of amyopathic DM (ADM) and hypomyopathic DM (HDM). Patients with other definite causes of interstitial lung disease, such as infectious pneumonia, chronic obstructive pulmonary disease (COPD), lung injury, and drug or occupational-environmental exposures were excluded at the initial diagnosis. Patients with complicating conditions, such as an active neoplasm and history of lung cancer, and other identifiable autoimmune diseases, such as systemic lupus erythematosus (SLE), rheumatoid arthritis (RA), or systemic sclerosis (SSc), or that had been treated with systemic corticosteroids and immunosuppressants before referral to our hospital were also excluded. This study was approved by the ethics committee of Peking University People's Hospital.

### Methods

Demographic, clinical, and laboratory data at the time of diagnosis and during follow-up were collected from hospital records. Demographic and clinical information, including age at onset, gender, disease duration at diagnosis, initial symptoms associated with the disease, Gottron's sign/papules, skin ulceration, periungual erythema, proximal muscle weakness, malignancy history, and ILD, were assessed. Laboratory data were recorded, including serum levels of creatine kinase (CK), aspartate aminotransferase (AST), lactate dehydrogenase (LDH), and ferritin. Myositis-specific autoantibodies (MSAs, antigens including Jo-1, PL-7, PL-12, EJ, OJ, KS, MDA5, NXP2, SAE, Mi-2, TIF-1γ) and myositis-associated autoantibodies (MAAs, antigens including Ro-52, PM-Scl, Ku) were identified in 207 patients by immunoblotting according to the manufacturers' instructions (Euroimmun, Germany).

Findings on arterial blood gas analysis, pulmonary function tests (PFT, including forced vital capacity, diffusing capacity for carbon monoxide and total lung capacity), chest high-resolution computed tomography (HRCT), and bronchoalveolar lavage fluid (BALF) were recorded at ILD diagnosis when available. Images of ILD on HRCT, including ground-glass attenuation (GGA), consolidations, nodular, reticulonodular, interlobular septal thickening, honeycombing, and traction bronchiectasis, were assessed. Based on the HRCT scan pattern, patients were classified into the following four groups: non-specific interstitial pneumonia (NSIP), lymphocytic interstitial pneumonia (LIP), usual interstitial pneumonia (UIP), and organizing pneumonia (OP). HRCT were reviewed by a panel of experienced radiologists according to 2013 ATS/ERS policies (18). The definition of RP-ILD was rapidly progressive dyspnea and hypoxemia with a worsening of radiologic interstitial lung changes within 3 months after the onset of respiratory symptoms. C-ILD was defined as an asymptomatic, slowly progressive ILD or as non-rapidly progressive over 3 months (19).

BALF was collected during bronchoscopy in clinic. Bronchoscopy was administrated with local anesthesia induced by lidocaine; 100 ml of sterile saline (0.9% NaCL) was instilled through the bronchoscope into the right lung field in two to four aliquots. BALF was collected after administration. Cellular components were separated from BALF by centrifugation (10 min, 1,200 rpm). Cytospin slides of cells in BALF were stained with hematoxylin-eosin for subsequent cell identification. The numbers of macrophages, lymphocytes, and neutrophils were recorded. The data of cytological analyses of BALF were collected from the standardized case record form in the clinical record. The R Maximal Selected Rank (MaxStat) package was used to determine the optimal cutoff point in lymphocytes in BALF to predict poor survival of RP-ILD.

### Statistical Analysis

Categorical variables were presented as frequency (percentages). Continuous data were expressed as the mean ± standard error or medians (interquartile range), and data on RP-ILD vs. C-ILD were compared using Student's *t*-test or the Mann–Whitney *U* test. Categorical variables were compared using Fisher's exact test or chi-square test. Outcomes were compared between RP-ILD patients and C-ILD patients. Survival between various groups was analyzed using a Kaplan–Meier curve with log rank test. Univariate and multivariate Cox regression analyses were used to identify predictors of poor survival due to RP-ILD.

## Results

### Characteristics of ILD in Patients With PM/DM/CADM

The study cohort included 505 patients with myositis and 31 patients with other autoimmune diseases (11 patients overlapped with SLE, 9 patients overlapped with SSc, 9 patients overlapped with RA, 2 patients overlapped with SLE+SSc) were excluded. A total of 474 patients with PM/DM/CADM were enrolled in this study, including 87.6% (369/474) females with a mean age of 49.7 ± 14.0 years (Table 1). ILD was found in 65% (308/474) of patients with PM/DM/CADM. ILD was identified to precede IIM clinical manifestations in 10.7% (33/308) of patients; among these patients with isolated ILD, 57.6% (19/33) of them developed myositis within 1 year after ILD diagnosis, 36.4% (12/33) were diagnosed with myositis 1–3 years after ILD diagnosis, and 6.1% (2/33) had myositis after 3 years. ILD onset was identified concurrently with PM/DM/CADM in 57.1% (176/308) of patients and occurred after IIM onset in 32.1% (99/308) of patients. Patients with ILD were divided into two groups according to pulmonary manifestations: RP-ILD (38%, 117/308) and C-ILD (62%, 191/308). The most common pattern of chest HRCT in IIM with ILD was NSIP (67.2%, 207/308), followed by OP (26.0%, 80/308) and UIP (6.8%, 21/308).

**Table 1:** Demographics and pulmonary characteristics of 474 patients with IIM.

**Variables**	***n* = 474**
Female, no. (%)	369 (87.6)
Age at onset, years	49.7 ± 14.0
**DIAGNOSIS**	
DM, no. (%)	216 (45.6)
CADM, no. (%)	201 (42.4)
PM, no. (%)	57 (12.0)
ILD, no. (%)	308 (65)
Rapidly progressive ILD, no. (%)	117/308 (38.0)
Chronic ILD, no. (%)	191/308 (62.0)
**ILD ONSET**	
Before IIM onset, no. (%)	33/308 (10.7)
Concomitant with IIM, no. (%)	176/308 (57.1)
After IIM onset, no. (%)	99/308 (32.1)
**HRCT PATTERN**	
NSIP, no. (%)	207/308 (67.2)
OP, no. (%)	80/308 (26.0)
UIP, no. (%)	21/308 (6.8)

*Continuous data are presented as M (mean) ± SEM (standard error of the mean). Binary data are presented as n/total number (percentage) of the patients. IIM, idiopathic inflammatory myositis; ILD, interstitial lung disease; DM, dermatomyositis; PM, polymyositis; CADM, clinically amyopathic dermatomyositis; HRCT, high resolution computerized tomography; NSIP, nonspecific interstitial pneumonia; OP, organizing pneumonia; UIP, usual interstitial pneumonia*.

### Clinical and Laboratory Features in IIM Patients With RP-ILD Compared With C-ILD

Among 117 consecutive patients with RP-ILD, 41% (48/117) of patients had DM, 51.3% (60/117) of patients had CADM, and 7.7% (9/117) of patients had PM (Table 2). Patients with RP-ILD were older than those with C-ILD (54.1 ± 12.7 vs. 50.1 ± 12.9 years, *P* = 0.009). The mean disease duration in the RP-ILD group was significantly shorter than the C-ILD group (2.0 ± 0.9 vs. 31.6 ± 59.4 months, *P* = 0.000). Additionally, fever, periungual erythema, skin ulceration, and subcutaneous/mediastinal emphysema were significantly more common in patients with RP-ILD compared with C-ILD with incidence rates of 63.2 vs. 37.2%, 22.2 vs. 12.0%, 11.1 vs. 3.1%, and 6.0 vs. 0.0%, respectively. The levels of serum LDH (*P* = 0.014), AST (*P* = 0.029), CRP (*P* = 0.019), and ferritin (*P* = 0.001) were significantly higher in the RP-ILD group than in the C-ILD group. Muscle weakness and malignancy were less common in patients with RP-ILD than those with C-ILD with incidence rates of 47.9 vs. 64.9% (*P* = 0.003) and 3.4 vs. 9.4% (*P* = 0.047). Moreover, peripheral blood lymphocytes were significantly lower in patients with RP-ILD compared with C-ILD (1.1 ± 0.7 vs. 1.5 ± 0.9, *P* = 0.000).

**Table 2:** Comparison of clinical and laboratory characteristics between DM/CADM/PM patients with RP-ILD and C-ILD.

**Variables**	**RP-ILD *n* = 117**	**C-ILD *n* = 191**	***P*-value**
**DIAGNOSIS**			
DM, no. (%)	48 (41.0)	79 (41.4)	0.954
CADM, no. (%)	60 (51.3)	88 (46.1)	0.375
PM, no. (%)	9 (7.7)	24 (12.6)	0.180
**DEMOGRAPHICS**			
Female, no. (%)	87 (74.4)	145 (75.9)	0.758
Age at onset, years	54.1 ± 12.7	50.1 ± 12.9	0.009^*^
Duration of ILD, months	2.0 ± 0.9	31.6 ± 59.4	0.000^*^
**CLINICAL VARIABLES**			
Fever, no. (%)	74 (63.2)	71 (37.2)	0.000^*^
Gottron's sign/papules, no. (%)	81 (69.2)	137 (71.7)	0.640
Periungual erythema, no. (%)	26 (22.2)	23 (12.0)	0.018^*^
Skin ulceration, no. (%)	13 (11.1)	6 (3.1)	0.005^*^
Muscle weakness, no. (%)	56 (47.9)	124 (64.9)	0.003^*^
Subcutaneous/mediastinal emphysema, no. (%)	7 (6.0)	0 (0.0)	0.001^*^
Malignancy, no. (%)	4 (3.4)	18 (9.4)	0.047^*^
**LABORATORY FEATURES**			
Lymphocytes, × 10^9^/L	1.1 ± 0.7	1.5 ± 0.9	0.000^*^
CK, U/L	65 (30.5,274.5)	72 (34,563)	0.448
LDH, U/L	324 (221,501)	281 (193.8,395)	0.014^*^
AST, U/L	38 (21.5,84.5)	30 (20,60)	0.029^*^
CRP, mg/dL	7.6 (2.4,31.0)	5.0 (1.9,13.0)	0.019^*^
Ferritin (ng/mL)^a^	1,065 (584.1,2690)	307.9 (129.8,881.3)	0.001^*^
Elevated CEA, no. (%)	37 (31.6)	22 (11.5)	0.000^*^
Elevated NSE, no. (%)	60 (51.2)	70 (36.6)	0.012^*^
Elevated CYFRA21-1, no. (%)	78 (66.7)	73 (38.2)	0.000^*^

*Continuous data were expressed as the mean ± standard error or medians (interquartile range). Binary data were presented as n (percentage) of the patients*.

a 49 patients of 117, 68 values missing in RP-ILD group;

* *< 0.05. IIM, idiopathic inflammatory myopathy; ILD, interstitial lung disease; RP-ILD, rapidly progressive ILD; C-ILD, Chronic ILD; CK, creatine kinase; LDH, lactate dehydrogenase; AST, aspartate aminotransferase; CRP, C-reactive protein, CEA, carcinoembryogenic antigen; NSE, neuron-specific enolase; CYFRA21-1, cytokeratin-19 fragment*.

In addition, increased CEA, NSE, and CYFRA21-1 in serum were significantly more common in the RP-ILD group than in the C-ILD group with incidence rates of 31.6 vs. 11.5%, 51.2 vs. 36.6%, and 66.7 vs. 38.2%, respectively. On the other hand, tumor markers including AFP, CA199, and CA125 were also screened for IIM patients, and there were no significant differences in these tumor markers between the RP-ILD and C-ILD groups. A total of 66.7% of patients with RP-ILD and 38.2% of patients with C-ILD had at least one of the tumor markers elevated in serum.

### Comparison of MSAs/MAAs in IIM Patients With RP-ILD and C-ILD

MSAs/MAAs were detected in 207 patients with ILD in the present study. Prevalence of anti-MDA5 and anti-Ro-52 antibodies were significantly higher in IIM patients with RP-ILD than with C-ILD with respective incidence rates of 39.0 vs. 12.0% (*P* = 0.000) and 58.5 vs. 40.8% (*P* = 0.012) (Table 3). Anti-ARS antibodies, especially anti-Jo-1 antibody (13.4 vs. 32.0%, *P* = 0.002) were detected less commonly in patients with RP-ILD compared with patients with C-ILD. There were no significant differences in prevalence of anti-Mi-2, anti-NXP2, anti-SAE, and other MAAs between the two groups. Out of 207 patients in which MSAs/MAAs were detected, 20 patients were identified without specific, associated myositis antibodies. Among these patients, ANA, RF, anti-SSA, anti-Sm, anti-Scl-70, anti-U1RNP, and ANCA were found in 35% (7/20), 20% (4/20), 5% (1/20), 0% (0/20), 0% (0/20), 5% (1/20), and 5% (1/20) of the patients, respectively.

**Table 3:** Comparison of MSAs/MAAs between IIM patients with RP-ILD and C-ILD.

**Variables**	**RP-ILD *n* = 82**	**C-ILD *n* = 125**	***P-*value**
**MYOSITIS-SPECIFIC ANTIBODIES**			
Anti-synthetase antibodies (+), no. (%)	35 (42.7)	71 (56.8)	0.047^*^
Anti-Jo-1, no. (%)	11 (13.4)	40 (32.0)	0.002^*^
Anti-MDA5, no. (%)	32 (39.0)	15 (12.0)	0.000^*^
Anti-Mi-2, no. (%)	2 (2.4)	3 (2.4)	1.000
Anti-TIF1-γ, no. (%)	3 (3.7)	4 (3.2)	1.000
Anti-NXP2, no. (%)	2 (2.4)	4 (3.2)	1.000
Anti-SAE, no. (%)	2 (2.4)	3 (2.4)	1.000
**MYOSITIS-ASSOCIATED ANTIBODIES**			
Anti-Ro-52, no. (%)	48 (58.5)	51 (40.8)	0.012^*^
Anti-PM/Scl-75/100, no. (%)	8 (9.8)	15 (12.0)	0.615
Anti-Ku, no. (%)	3 (3.7)	7 (5.6)	0.743

* *< 0.05. IIM, idiopathic inflammatory myopathy; ILD, interstitial lung disease; RP-ILD, rapidly progressive ILD; C-ILD, Chronic ILD. ARS include EJ, OJ, PL-7, PL-12, KS. ARS, aminoacyl-tRNA synthetase; MDA5, melanoma differentiation-associated 5; TIF-1γ, translation initiation factor-1a; NXP2, nuclear matrix protein 2; SAE, small ubiquitin-like modifier enzyme; PM/Scl, polymyositis/scleroderma*.

### Pulmonary Characteristics and Mortality of IIM Patients With RP-ILD and C-ILD

OP pattern on HRCT was more common in the RP-ILD group than in the C-ILD group at the initial assessment with incidence rates of 52.1 vs. 11.0% (*P* = 0.000) (Table 4). In contrast, NSIP and UIP patterns were associated with C-ILD as the incidence rates were 47.9 and 0.0% in RP-ILD subjects compared to 78.0 and 11% in C-ILD subjects, respectively. In total, 161 patients finished PFT and arterial blood gas analysis at initial evaluation, and these results were consistent with ILD in all patients. The results of decreased PaO_2_ (*P* = 0.000) and PFTs, including lower FVC (*P* = 0.000), DL_CO_ (*P* = 0.000), and TLC (*P* = 0.000) verified severe lung impairment in patients with RP-ILD compared with those with C-ILD. Analysis of cell composition in BALF showed a significantly increased proportion of lymphocytes and decreased macrophage cells in the RP-ILD group compared with the C-ILD group with rates of 38.2 ± 23.2 vs. 20.4 ± 13.1 (*P* = 0.000) and 47.9 ± 22.5 vs. 68.8 ± 16.1 (*P* = 0.000). Out of 117 patients with RP-ILD, 78 received bronchoalveolar lavage immune cell tests, including 12 patients that did not survive and 66 that survived. Lymphocytes in BALF at <30% was found in 83.3% (10/12) of deceased patients compared with only 33.3% (22/66) of patients who survived (*P* = 0.003) (Supplementary Table S1). Out of 191 patients with C-ILD, 97 received bronchoalveolar lavage tests. Lymphocytes in BALF at <30% was found in 100% (7/7) of deceased patients with C-ILD compared with 81.1% (73/90) of C-ILD patients that survived, but the difference was not significant (*P* = 0.348) (Supplementary Table S2).

**Table 4:** Comparison of baseline pulmonary features and initial treatment between IIM patients with RP-ILD and C-ILD.

**Variables**	**RP-ILD *n* = 117**	**C-ILD *n* = 191**	***P-*value**
PaO_2_ < 80 (mmHg)^a^	59 (92.2)	22 (22.7)	0.000^*^
**BASELINE PFTs (% PREDICTED)**^**a**^			
FVC	65.7 ± 16.2	86.9 ± 15.1	0.000^*^
DLco	48.5 ± 16.0	72.3 ± 16.2	0.000^*^
TLC	70.6 ± 15.5	88.2 ± 14.4	0.000^*^
**HRCT PATTERN**			
NSIP, no. (%)	56 (47.9)	149 (78.0)	0.000^*^
OP, no. (%)	61 (52.1)	21 (11.0)	0.000^*^
UIP, no. (%)	0 (0.0)	21 (11.0)	0.000^*^
**BRONCHOALVEOLAR LAVAGE**^**b**^			
Total cell number (×10^5^/ml)	3.0 ± 2.9	3.1 ± 3.2	0.137
Macrophage (%)	47.9 ± 22.5	68.8 ± 16.1	0.000^*^
Lymphocyte (%)	38.2 ± 23.2	20.4 ± 13.1	0.000^*^
Neutrophil (%)	12.6 ± 18.3	9.1 ± 10.0	0.084
Mortality, no. (%)	32 (27.4)	15 (7.9)	0.000^*^
Median time to death, years	0.2 (0.1, 1.5)	5.7 (1.0, 10.1)	0.012^*^
**CAUSE OF DEATH**			
Respiratory failure, no. (%)	20 (62.5)	2 (13.3)	0.002^*^
RF complicated with infection, no. (%)	8 (25.0)	1 (6.7)	0.236
Cancer, no. (%)	0 (0.0)	6 (40.0)	0.000^*^
Others, no. (%)	4 (12.5)	6 (40.0)	0.054
**INITIAL TREATMENT**			
CS pulse therapy (0.5 g/d IV 3 days)	103 (88.0)	15 (7.9)	0.000^*^
**IMMUNOSUPPRESSANTS**			
CsA	38 (32.5)	22 (11.5)	0.000^*^
MMF	1 (0.9)	12 (6.3)	0.020^*^
Tac	3 (2.6)	1 (0.5)	0.155
Intravenous CYC	74 (63.2)	90 (47.1)	0.007^*^
CsA+CYC	8 (7.3)	1 (0.5)	0.002^*^
Tofacitinib	4 (3.4)	0 (0.0)	0.020^*^
Rituximab	2 (1.7)	0 (0.0)	0.144

*Data are presented as n (percentage) of the patients. Data of pulmonary function test and bronchoalveolar lavage are presented as mean ± SEM*.

* *< 0.05*.

a *64 patients of 117, 53 values of baseline PaO_2_, FVC, DLco, TLC missing in RP-ILD group; 97 patients of 191, 94 values of baseline PaO_2_, FVC, DLco, TLC missing in C-ILD group*.

b *78 patients of 117, 39 values of bronchoalveolar lavage immune cell tests missing in RP-ILD group; 97 patients of 191, 94 values of bronchoalveolar lavage immune cell tests missing in C-ILD group. IIM, idiopathic inflammatory myopathy; ILD, interstitial lung disease; RP-ILD, rapidly progressive ILD; C-ILD, chronic ILD; HRCT, high resolution computerized tomography; NSIP, non-specific interstitial pneumonia; UIP, usual interstitial pneumonia; OP, organizing pneumonia; FVC, forced vital capacity; DLco, diffusion capacity for carbon monoxide; TLC, total lung capacity; RF: respiratory failure; IV, intravenous injection; CS, glucocorticoid; CsA, Cyclosporine; MMF, Mycophenolate mofetil; CYC, Cyclophosphamide; Tac, Tacrolimus*.

The mortality rates in patients with RP-ILD were significantly higher than those in the C-ILD group (27.4 vs. 7.9%, *P* = 0.000, respectively). The median time to death was 0.2 years in RP-ILD subjects compared to 5.7 years in C-ILD subjects. The main cause of death in the RP-ILD group was respiratory failure due to RP-ILD (62.5%, 20/32), and a quarter of patients died from complicating infections. We also compared therapeutic data between the two groups (Table 4). Patients in the RP-ILD group received more aggressive initial treatment regimes compared with patients in the C-ILD group. A total of 88% of patients with RP-ILD were treated with CS pulse therapy compared with 7.9% of patients with C-ILD at initial treatment (*P* = 0.000). Calcineurin inhibitors, especially cyclosporine, and intravenous cyclophosphamide (0.4–0.6 g every 2 weeks) were preferentially used in the RP-ILD group rather than mycophenolate mofetil; rituximab, tacrolimus, and tofacitinib were seldom used.

### Survival Analysis of IIM Patients With RP-ILD

**Figure 1:**
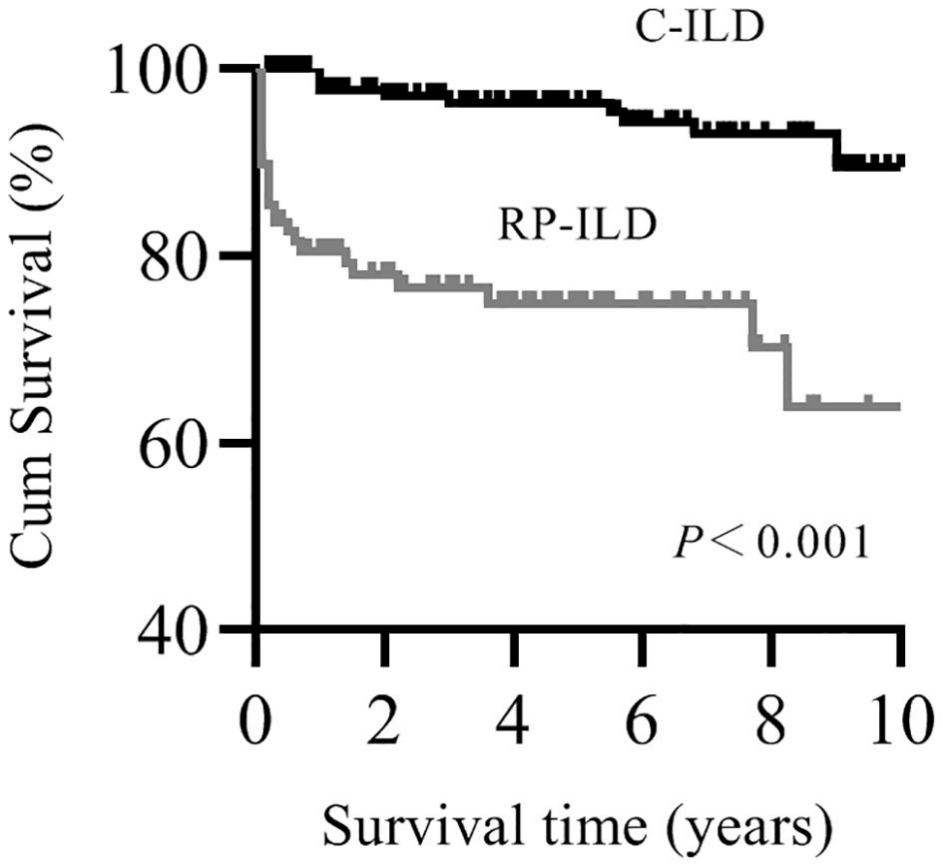
Kaplan–Meier survival curves for myositis-associated RP-ILD and C-ILD.

**Figure 2:**
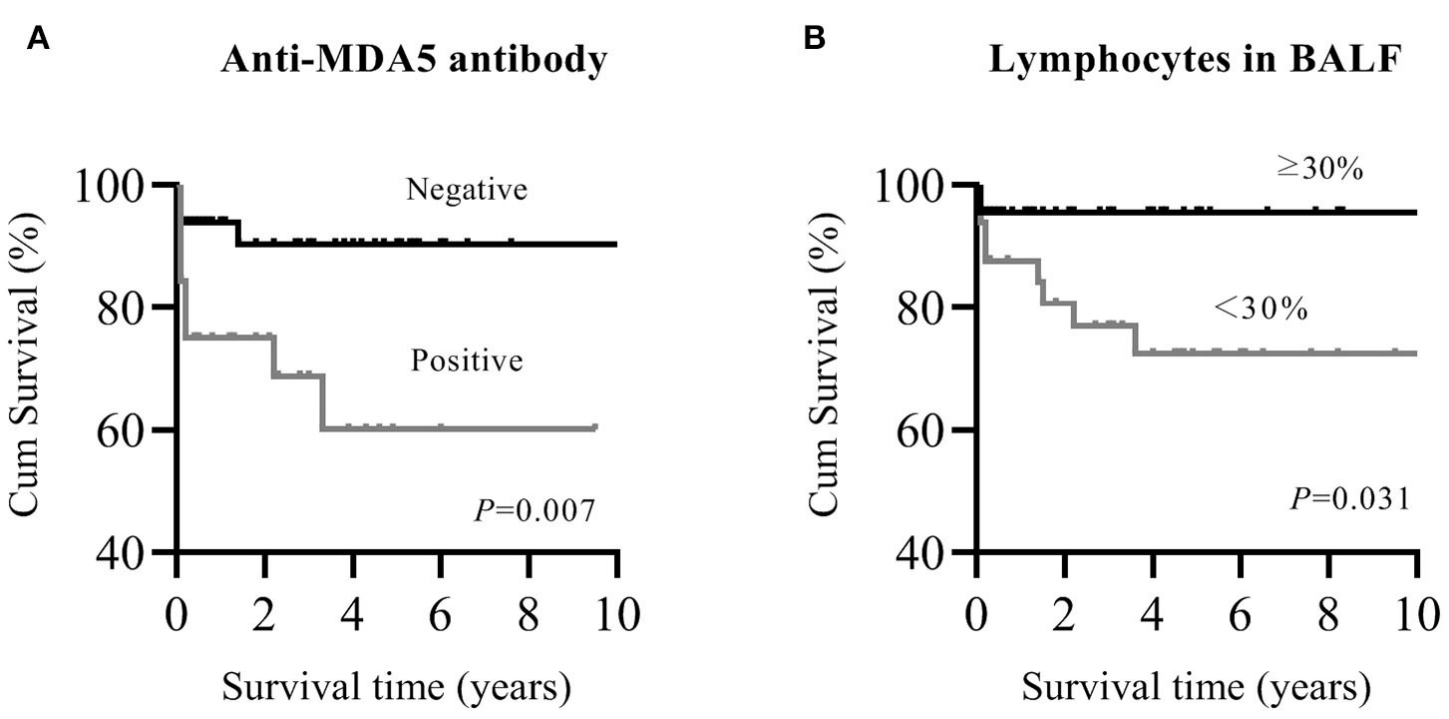
Kaplan–Meier survival curves for myositis-associated RP-ILD. **(A)**, MDA5 positive and MDA5 negative; **(B)**, lymphocytes in BALF <30% and ≥30%. MDA5, melanoma differentiation-associated 5; RP-ILD, rapidly progressive interstitial lung disease; BALF, bronchoalveolar lavage fluid.

Patients with myositis-associated RP-ILD had significantly lower survival rates than the C-ILD group (1-year survival, 76 vs. 98%; 5-year survival, 73 vs. 94%; *P* = 0.000) (Figure 1). Moreover, skin ulceration, LDH > 245 U/L, AST > 40 U/L, lymphocytes in BALF <30%, and anti-MDA5 antibody were associated with mortality on univariate analysis. Multivariate Cox proportional hazards model analysis identified that anti-MDA5 antibody (HR 11.639, [95% CI 1.338–101.240], *P* = 0.026) was an independent risk factor for mortality due to RP-ILD, and lymphocytes at <30% in BALF (HR 12.048, [95% CI 1.466–99.031], *P* = 0.021) might be associated with poor survival of RP-ILD (Table 5). Among patients with RP-ILD, anti-MDA5-positivity was significantly associated with poor survival (57% at both 5 and 10 years) compared to the anti-MDA5-negative group (89% at both 5 and 10 years, *P* = 0.007) (Figure 2A). Additionally, lymphocytes <30% in BALF might also be associated with poor survival of RP-ILD (87.3% at 5 years and 80.3% at 10 years) compared with lymphocytes at ≥30% in BALF (95.7% at both 5 and 10 years, *P* = 0.031) (Figure 2B). Notably, due to lack of data in BALF tests (33.3% in RP-ILD group and 49% in C-ILD group), the statistical power of analysis of the BALF lymphocyte ratio was insufficient, and a probable selection bias existed. Therefore, this result needs to be validated in future studies.

**Table 5:** Survival analysis in myositis-associated RP-ILD (Cox proportional hazards model).

**Variables**	**Hazard ratio**	**95% CI**	***P*-value**
***UNIVARIATE***			
Fever	2.823	0.730–10.918	0.133
Skin ulceration	3.726	1.554–8.932	0.003^*^
Subcutaneous/mediastinal emphysema	2.999	0.721–12.475	0.131
LDH > 245 U/L	1.001	1–1.001	0.001^*^
AST > 40 U/L	1.005	1.002–1.008	0.002^*^
Anti-Jo-1 antibody	0.040	0–8.705	0.040^*^
Anti-MDA5 antibody	11.320	1.450–88.356	0.021^*^
Lymphocytes in BALF <30%	5.281	1.133–24.623	0.034^*^
***MULTIVARIATE***			
Anti-MDA5 antibody	11.639	1.338–101.240	0.026^*^
Lymphocytes in BALF <30%	12.048	1.466–99.031	0.021^*^
Skin ulceration	1.283	0.240–6.863	0.770

* *< 0.05. Initial predictors for poor survival of myositis-associated RP-ILD due to respiratory failure were verified by multivariate analysis. MDA5, melanoma differentiation-associated 5; RP-ILD, rapidly progressive interstitial lung disease; BALF, bronchoalveolar lavage fluid*.

## Discussion

RP-ILD, a common complication of IIM, is a poor prognostic factor for patients with IIM (4, 5). Therefore, these patients need careful evaluation of clinical characteristics and radiological features during follow-up (20). The present study retrospectively reviewed 474 cases of IIM and identified initial predictors for myositis-associated RP-ILD from an inpatient rheumatology cohort in China.

The prevalence of ILD was 65% in patients with DM/PM/CADM, and nearly 40% of them had RP-ILD in our center. The prevalence of ILD in our center is higher than other historical series (21). The possible reason is that our hospital is a well-known center for myositis and other rheumatic diseases in China, so increased frequency of severe patients with ILD were found in the in-patient clinical records. In addition, all patients received routine examination of HRCT to screen for potential ILD, which might lead to a higher prevalence of ILD in this cohort. However, differences might also exist in different countries. According to several other cohort studies, it seems that the prevalence of ILD in our study was similar with these previous studies and was not extraordinary (22, 23). The present study showed 10.7% of patients diagnosed with ILD before the diagnosis of IIM, so these patients required intensive evaluation during follow-up to reduce the rate of misdiagnosis. NSIP on chest HRCT of IIM patients was reported to be the most common pattern in our study, and this result was consistent with previous studies (24, 25).

Previous studies have identified that survival rates of patients with myositis-associated RP-ILD were lower than in C-ILD (26). Won et al. (27) report a 3-year survival rate for RP-ILD of 27.3%, and Fujisawa et al. (28) report a 5-year survival rate of 52% in the RP-ILD. However, the 5-year survival rate of the RP-ILD group in our study was 73%, which is higher than in previous reports. The potential reason may be the choice of different treatment regimens or different therapeutic effects among racial types. Rapid deterioration and infection secondary to over-immunosuppression were two main causes of death, so appropriate therapy regimens still need to be pursued by clinicians.

This study verified many clinical and laboratory prognostic factors previously reported to be associated with RP-ILD in IIM patients, such as age at onset, fever, periungual erythema, skin ulceration, and decreased peripheral blood lymphocyte cells as well as increased levels of AST, ferritin, LDH, and CRP (29). Additionally, serum tumor markers, such as CEA, NSE, and CYFRA21-1 were found to be associated with RP-ILD in our study. Although such tumor markers have been used to screen potential cancer in clinical practice, this result has not been reported before. The possible reason is that these tumor markers could be induced by intensive inflammation in lung.

Measurement of MSAs and MAAs are helpful in classifying different subtypes of IIM in clinical practice. Our study demonstrated that anti-MDA5 antibody was a specific biomarker for myositis-associated RP-ILD. Anti-Ro-52 antibody was also associated with RP-ILD in our study. These findings were consistent with previous studies (25, 30–32). In contrast, anti-ARS antibodies, especially anti-Jo-1 antibody, were related to myositis-associated C-ILD in our study, which indicated that anti-ARS antibodies may be a favorable predictor for RP-ILD. The multivariate Cox proportional hazards model analysis used in our study identified anti-MDA5 antibody as an independent predictor of poor outcome in patients with myositis-associated RP-ILD. The importance of anti-MDA5 antibody in the prognosis of myositis has been described by Tanizawa et al. (16), who showed that anti-MDA5 was an independent determinant of overall mortality in DM/PM patients with ILD.

Our analysis verified that low PaO_2_, FVC, DL_CO_, and TLC were associated with RP-ILD. This result confirmed that analyzing arterial blood gas and PFT were useful tests for myositis-associated RP-ILD. FVC and DL_CO_ values have been reported as predictive factors for poor prognosis of ILD in IIM (33, 34). Our study also found that initial low TLC was correlated with the onset of RP-ILD.

Currently, cellular profiles in BALF are used in patients with myositis to rule out infection in clinical practice. The relationship between cellular profiles of BALF and poor prognosis has not been supported by all studies (28, 35). Schnabel et al. (35) report the presence of neutrophils in BALF associated with progressive ILD. In contrast, Fujisawa et al. (28) indicate that a relatively high percentage of lymphocytes in BALF is correlated with myositis-associated ILD. However, our study demonstrates increased lymphocyte infiltration and decreased number of macrophage cells in BALF are associated with onset of RP-ILD in myositis patients. Our study further shows that lymphocytes at <30% in BALF is probably associated with poor survival of myositis-associated RP-ILD. The ATS guidelines (36) indicate that the presence of >15% lymphocytes in BALF represents a lymphocytic cellular pattern such as OP or NSIP.

Takei et al. (37) report that corticosteroids and other immunosuppressants are more effective in the patients with a lymphocyte differential count >15% than in patients with a lymphocyte differential count <15%. According to Takei et al., we speculate that the reason for this association is that patients with a lower lymphocyte ratio in BALF might respond poorly to treatment with corticosteroids or immunosuppressants, which might lead to poorer outcomes. However, due to the rather high percentage of missing data in BALF results (33.3% in RP-ILD group and 49% in C-ILD group), the statistical power of analysis of BALF lymphocyte ratio is insufficient. Only 10.3% (12/117) of patients died in the subgroup of RP-ILD patients with available BALF results compared to the overall mortality of 27.4% (32/117), which suggests a probable selection bias. Therefore, this result needs to be validated in future studies. It should be noted that the cutoff level of lymphocytes <30% in BALF should also be validated in future studies. Further research on lymphocyte subsets and function is also needed in future work to elucidate the immunological mechanism of different lymphocyte phenotypes and functions in myositis-associated RP-ILD.

There are several limitations in the present study. The retrospective nature and the selection of cases from a single center might have caused a selection bias. Because patients were selected from a center for myositis and other rheumatic diseases, more severe forms of disease were recorded. Because the study was retrospective, follow-up time was different among the cases, and some missing data could not be avoided. For example, MSAs, MAAs, lung function, and BALF test (including subsets of lymphocytes) were not performed in all the patients. On the other hand, the strength of the study is that it includes a large cohort of patients with myositis who have undergone HRCT. Further prospective and multicenter studies are needed to overcome these weaknesses.

## Conclusions

Our study highlights that presence of RP-ILD results in an increased rate of mortality in DM/PM/CADM. IIM patients with predictive factors of RP-ILD, including anti-MDA5 antibody and lymphocytes <30% in BALF, should receive intensive follow-up.

## Data Availability Statement

All datasets generated for this study are included in the article/Supplementary Material.

## Ethics Statement

Written informed consent was obtained from the individual(s) for the publication of any potentially identifiable images or data included in this article.

## Author Contributions

YuL, JH, and XS conceived and designed the study and wrote the manuscript. XG, YiL, XJ, YG, YX, XZ, RC, and SL collected the data. YuL, XG, and YiL analyzed the data. All authors contributed to the article and approved the submitted version.

## Conflict of Interest

The authors declare that the research was conducted in the absence of any commercial or financial relationships that could be construed as a potential conflict of interest.
