# Supplementary material for: Predictors and Mortality of Rapidly Progressive Interstitial Lung Disease in Patients With Idiopathic Inflammatory Myopathy: A Series of 474 Patients
Source: Front Med (Lausanne). 2020 Jul 31;7:363. doi: 10.3389/fmed.2020.00363 (PMC7412929; doi:10.3389/fmed.2020.00363)
Supplement: Supplementary file 1 [file Table_1.pdf]

Table S1. Comparison of lymphocytes in BALF in survived and non-survived patients with RP-ILD

	RP-ILD (n=78/117)		*P*-value
	Survived<br>n=66/85	Non-Survived<br>n=12/32	
Lymphocytes in BALF<30% (n, %)	22 (33.3)	10 (83.3)	0.003*
Lymphocytes in BALF≥30% (n, %)	44 (66.7)	2 (16.7)	0.003*

*<0.05. RP-ILD, rapidly progressive interstitial lung disease; BALF, bronchoalveolar lavage fluid.

Table S2. Comparison of lymphocytes in BALF in survived and non-survived patients with C-ILD

	C-ILD (n=97/191)		*P*-value
	Survived<br>n=90/176	Non-Survived<br>n=7/15	
Lymphocytes in BALF<30% (n, %)	73 (81.1)	7 (100.0)	0.348
Lymphocytes in BALF≥30% (n, %)	17 (18.9)	0 (0.0)	0.348

C-ILD, chronic interstitial lung disease; BALF, bronchoalveolar lavage fluid.
